# The effect of aerobic exercise on tumour blood delivery: a systematic review and meta-analysis

**DOI:** 10.1007/s00520-022-07132-0

**Published:** 2022-06-02

**Authors:** Catherine Seet-Lee, Jasmine Yee, Heidi Morahan, Lois S. Ross, Kate M. Edwards

**Affiliations:** 1 Faculty of Medicine and Health, University of Sydney, Camperdown, NSW 2006 Australia; 2 Charles Perkins Centre, University of Sydney, Camperdown, Australia; 3 Centre for Medical Psychology & Evidence-Based Decision-Making, School of Psychology, The University of Sydney, Camperdown, Australia; 4 Department for Health, University of Bath, Bath, UK

**Keywords:** Exercise, Tumour, Hypoxia, Vascularisation, Blood flow

## Abstract

**Purpose:**

Tumour blood vessels are structurally and functionally abnormal, resulting in areas of hypoxia and heterogeneous blood supply. Aerobic exercise may modulate tumour blood flow and normalise the tumour microenvironment to improve chemotherapy delivery. This systematic review and meta-analysis aimed to evaluate the effect of the aerobic exercise mode on tumour hypoxia, vascularisation and blood flow.

**Methods:**

Four online databases were searched. Preclinical and clinical randomised controlled trials examining the effects of aerobic exercise training on hypoxia, vascularisation or blood flow in solid tumours were included. The risk of bias was assessed and a meta-analysis performed.

**Results:**

Seventeen preclinical studies and one clinical study met criteria. Eleven studies assessed hypoxia, 15 studies assessed vascularisation and seven evaluated blood flow. There was large variability in measurement methods, tumour types and exercise program designs. The overall risk of bias was unclear in clinical and preclinical studies, owing to poor reporting. There was no significant effect of aerobic exercise on hypoxia (SMD = −0.17; 95% CI = −0.62, 0.28; *I*^2^ = 60%), vascularisation (SMD = 0.07; 95% CI = −0.40, 0.55; *I*^2^ = 71%) or blood flow (SMD = 0.01; 95% CI = −0.59, 0.61; *I*^2^ = 63%).

**Conclusion:**

There is heterogeneity in methodology, resulting in evidence that is inconsistent and inconclusive for the effects of aerobic exercise on hypoxia, vascularisation and blood flow. Most evidence of aerobic exercise effects on tumour blood flow is in animal models, with very limited evidence in humans.

**Supplementary Information:**

The online version contains supplementary material available at 10.1007/s00520-022-07132-0.

## Introduction

Cancer incidence worldwide in 2016 was 17.2 million cases, with a 28% increase observed between 2006 and 2016 [1]. There are many modifiable risk factors for cancer development, including obesity and physical inactivity, which combined contribute to 25% of cancer incidence [2, 3]. Exercise during cancer treatment is recognised as an important adjunct to cancer treatment, with significant benefits on quality of life and a reduction in treatment side effects [4–7]. It has been proposed that exercise may also induce adaptations to tumour vasculature and thus stimulate benefit to clinical outcomes of cancer treatment [8].

Unlike in healthy tissues, where blood vessels typically run in parallel, blood vessels in tumours have an unstructured distribution. Tumour centres have high interstitial fluid pressure, leading to the collapse and abnormal compression of vasculature [8]. These structural and functional abnormalities lead to heterogeneous blood flow in in regions of the tumour which is further compounded by necrotic tumour centres [8]. The implication of these abnormal features results in a hypoxic tumour environment that is proposed to suppress immune function, limit the transport of immune cells to tumour regions and increase metastasis, leading to poorer patient prognosis [8–11].

Acute aerobic exercise increases total blood flow to healthy active tissue (e.g. contracting skeletal muscle) through the combination of increased cardiac output, increased blood pressure and local vessel vasodilation, with vasoconstriction reducing or maintaining blood flow to inactive tissues [8, 12]. Arterioles in tumours are poorly developed and lack functional smooth muscle [13] and subsequently have a reduced myogenic vasoconstriction response at high pressures such as during aerobic exercise [14]. This inability to vasoconstrict suggests that exercise-induced increased cardiac output, in addition to increased blood pressure, would drive an increase in tumour perfusion through increased total blood flow [14].

Repeated bouts of aerobic exercise cause vascular adaptions in healthy tissue. The combination of angiogenesis and decreased resistance enables increased blood flow to active healthy tissue [12]. These effects have been proposed to be paralleled in tumours, such that aerobic exercise training may cause adaptations that modulate tumour blood flow through increased blood vessel density and improved organisation and vessel function [8, 13]. If these adaptions occur, aerobic exercise training may normalise the tumour microenvironment and facilitate increased blood flow and reduced hypoxia, which may have benefits for reducing cancer progression. Given that many cancer treatments such as chemotherapy are administered intravenously, the aerobic exercise training effects of increased blood flow and improved vessel function have the additional potential for improving the delivery and therefore efficacy of such treatments [14].

The purpose of this systematic review is to: (i) summarise the effects of aerobic exercise training on tumour hypoxia, vascularisation and blood flow; and (ii) evaluate the methodological rigour of this literature.

## Methods

This systematic review and meta-analysis is reported in accordance with the Preferred Reporting Items for Systematic Reviews and Meta-Analysis (PRISMA) guidelines. This review was registered with the PROSPERO Register of Systematic Reviews (CRD42020159201).

### Eligibility criteria

Trials were included if they: (1) were a peer-reviewed journal full-text article in English; (2) used a randomised or quasi-randomised study design with a control group; (3) involved humans or animals with a solid malignant tumour; (4) the intervention group performed repeated bouts of any type of aerobic exercise of ≥2 sessions; (5) the control group performed no structured or unstructured aerobic exercise; (6) measured hypoxia, vascularisation, blood flow or indicators thereof.

### Search strategy and study selection

We searched Medline via OvidSP (1946–present), EMBASE via OvidSP (1947–present), Scopus (all years) and CINAHL Complete (all years) in September 2021 using the following search terms: ‘exercise’ OR ‘physical activity’ OR ‘exercise therapy’ OR ‘aerobic exercise’ AND ‘neoplasm’ OR ‘tumour’ OR ‘tumor’ OR ‘carcinoma’ OR ‘cancer’ OR ‘tumour microenvironment’ OR ‘tumor microenvironment’ OR ‘tumour vasculature’ OR ‘tumor vasculature’ AND ‘blood delivery’ OR ‘blood flow’ OR ‘vascularisation’ OR ‘vascular function’ OR ‘vascular remodeling’ OR ‘hypoxia’ OR ‘tumor hypoxia’ OR ‘tumour hypoxia’ OR ‘oxygenation’.

Two reviewers (CSL, JY) independently performed the initial screening by title and abstract based on the eligibility criteria. Full-text versions of potential eligible studies were then assessed by two reviewers independently (CSL, JY). A third reviewer (KE) screened full-text studies if there were disagreements between the researchers regarding eligibility.

### Outcome measures and data extraction

Two reviewers (CSL, LR) independently extracted data, and a third reviewer (KE) performed data extraction if there were disagreements between the researchers. Data were extracted for the three variables; hypoxia, vascularisation and blood flow. Hypoxia included any measure indicative of hypoxia as identified by the study author. Vascularisation includes microvessel density and changes in vessel physiology (including number of functional and patent vessels). Blood flow included changes in both tumour vessel perfusion and tissue perfusion (by MRI or Hoechst 33342 staining). Data extracted were recorded as the change in mean values from baseline for each group, and the standard error of the mean (SMD), standard deviation (SD) or confidence interval (CI) was also recorded. Study characteristics extracted included study details and design, recruitment source and method, participant results, experimental protocol, adherence and funding.

### Quality assessment

The included studies were assessed for internal validity using SYRCLE’s Risk of Bias Tool [15] or Cochrane Risk of Bias Tool [16]. The SYRCLE Risk of Bias Tool is used to determine the internal validity of animal studies. The tool contains 10 questions relating to 6 different domains of bias; selection bias, performance bias, detection bias, attrition bias, reporting bias and other biases. Each entry was scored as ‘no’ (indicating high risk), ‘yes’ (indicating low risk) or ‘unclear’ (indicating unclear risk). Each study was assessed in its entirety, irrespective of whether the study had multiple outcome measures. Baseline characteristics included age, sex, tumour type, site of tumour injection and timing of tumour induction prior to randomisation. Studies had to explicitly report study characteristics to assess the risk of bias. Housing allocation was also assessed in Domain 10, specifically if animals were housed individually and analysed individually.

The Cochrane Risk of Bias Tool is used to determine the internal validity of human studies [16]. The tool contains 5 questions that each covers a domain of bias; selection bias, performance bias, detection bias, attrition bias and reporting bias. Within each domain are signalling questions that draw relevant information from the study to determine risk of bias. The responses to the signalling questions are formulated in an algorithm to determine a judgement of ‘low risk’, ‘some concern’ or ‘high risk’. The Risk of Bias Tool was used for each outcome measure if the study included multiple outcome measures.

Two researchers (CSL, LR) independently performed these assessments. A third reviewer (JY) performed an assessment if there were disagreements between the researchers.

## Statistical analysis

A meta-analysis was performed for hypoxia, vascularisation and blood flow. Data from the outcome measures and indicators thereof were extracted as the mean and standard deviation. Data that were presented as SEM or CI were converted to SD. Data were included if they were quantitative and physiologically plausible. Relative values and fold changes were excluded if raw data were unable to be obtained. Negative values, such as for changes in hypoxia-inducible factor 1-alpha (HIF1α), were excluded as these are not physiologically feasible. For studies that had a range in sample size, the lower end of the sample size was used to avoid overpowering the study. The raw data can be found in the Supplemental materials (Supplemental Table 1).

The meta-analysis was performed in RStudio (R version 4.0.3) using a random-effects model. Heterogeneity was assessed by *I*^2^, and a subgroup analysis was performed on hypoxia, vascularisation and blood flow for animals, species, aerobic exercise mode, tumour location and study duration. The meta-analysis was controlled for the inclusion of multiple datasets that were compared to the same control group within a single study using the following equation; *N*_corrected control_ = *N*_control_ / number of experimental groups [17].

## Results

### Study selection

**Fig. 1:**
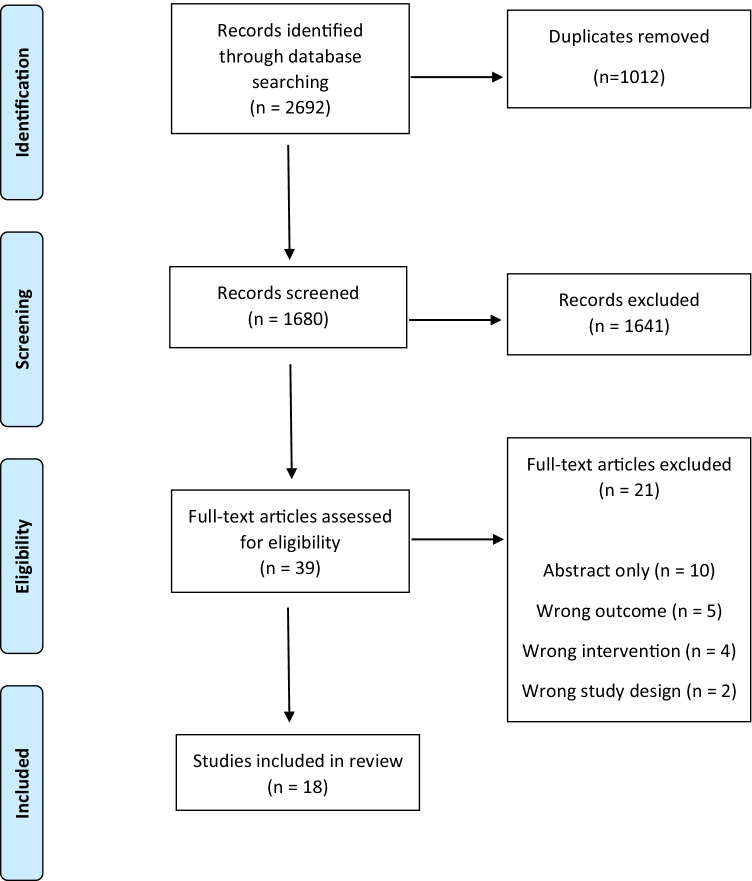
PRISMA flowchart

The initial database searches yielded 2692 studies (Fig. 1). After duplicates were removed, a total of 1680 studies were screened by title and abstract. Thirty-nine full-text articles proceeded for further review. A total of 18 studies were deemed suitable for inclusion in this review.

### Study and participant characteristics

Details of study and participant characteristics of the included studies are presented in Table 1. Of the 18 studies included, 17 were preclinical and one was clinical. Only one study was published prior to 2010. Multiple studies assessed more than one outcome measure; 11 assessed hypoxia, 15 assessed vascularisation and seven evaluated blood flow. Of the 17 preclinical studies, 14 used mice and four used rats. Study duration ranged from five to 245 days. Aerobic exercise in the preclinical studies included both voluntary (*n* = 6) and forced exercise (*n* = 11). The six studies that used voluntary aerobic exercise adopted wheel running, performed daily or every second day. The remaining 11 studies used treadmill running, of which nine studies were five times per week, two studies were daily and one study was every second day. One study used two different aerobic exercise frequencies in different groups. One clinical study explored cycle training sessions three times per week at 55–100% VO_2peak_ in women with breast cancer.

**Table 1:** Study characteristics

Variable	Preclinical trials (*n* = 17)	Clinical trials (*n* = 1)
Publication year		
2000–2009	1 (6)	0 (0)
2010–2021	16 (94)	1 (100)
Sample size		
≤20	7 (41)	1 (100)
21–49	7 (41)	0 (0)
≥50	3 (18)	0 (0)
Study duration		
<3 weeks	7 (41)	0 (0)
3–8 weeks	8 (47)	0 (0)
>8 weeks	2 (12)	1 (100)
Animal species^a^		
Mice	14 (82)	–
Rat	4 (24)	–
Animal breed/strain		
BALB/c mice	5 (29)	–
Nude mice^a^	3 (18)	–
C57BL/6 mice	3 (18)	–
Sprague-Dawley rat	2 (12)	–
Athymic mice	2 (12)	–
ApoE−/− mice	1 (6)	–
Copenhagen rats^a^	1 (6)	–
American Cancer Institute rats	1 (6)	–
Cancer type		
Breast^b^	9 (53)	1 (100)
Prostate	3 (18)	0 (0)
Pancreatic^b^	2 (12)	0 (0)
Melanoma^b^	2 (12)	0 (0)
Ewing sarcoma^b^	1 (6)	0 (0)
Liver	1 (6)	0 (0)
Lymphatic	1 (6)	0 (0)
Exercise mode		
Type		
Treadmill running	11 (65)	0 (0)
Wheel running	6 (35)	0 (0)
Cycling	0 (0)	1 (100)
Frequency^c^		
1–4×/week	1 (6)	1 (100)
5–7×/week	17 (100)	0 (0)
Outcome measure^d^		
Hypoxia	10 (59)	1 (100)
Vascularisation	14 (82)	1 (100)
Blood flow	6 (35)	1 (100)

Data are presented as *n* (%)

^a^Some studies included more than one animal species and breed

^b^Some studies included more than one cancer type

^c^Some studies included 2 groups that performed different exercise frequencies

^d^Some studies included more than one outcome measure

### Risk of bias

#### Preclinical: SYRCLE Risk of Bias

**Fig. 2:**
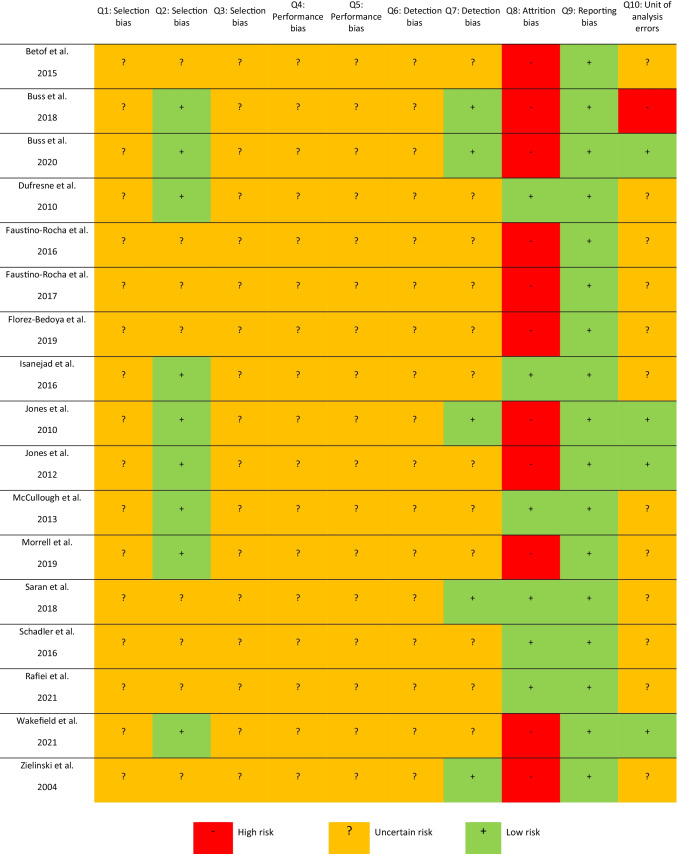
SYRCLE Risk of Bias for preclinical studies

The risk of bias across the preclinical studies is reported in Fig. 2. Of concern, more than half of the included studies (*n* = 11) had a high risk of attrition bias due to not reporting missing data appropriately. However, all 17 studies were free of selective outcome reporting as all available results were presented as described in the study methods. Nine studies reported clear baseline characteristics and therefore were assessed as having a low risk, while the remaining eight studies did not clearly report all baseline characteristics and were assessed as having an unclear risk. Most other outcomes were assessed as having an unclear risk due to poor reporting of study methodology.

#### Clinical: Cochrane Risk of Bias

**Fig. 3:**
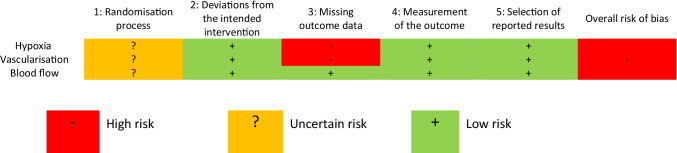
Cochrane Risk of Bias for clinical studies

Overall, there was a high risk of bias for the clinical study, primarily a result of missing outcome data for hypoxia and vascularisation (Fig. 3). There were additional concerns regarding randomisation, as the study did not detail the concealment procedure. Blinding of participants was not possible due to the pragmatic nature of the exercise intervention, although there was still low risk of bias for deviations from the intended intervention. Although the overall risk of bias was high, there was low risk of bias in the measurement and reporting of all outcomes (Fig. 3).

**Table 2:** Summary of studies

Author (year)	Participants	Housing condition	Cancer type/model	Intervention	Outcome measure: method of measure	Author reported result	Other comments
Preclinical studies							
Betof et al. (2015)	*N* = 22–24 female immunocompetent BALB/c mice	Housing groups: control group were housed individually; experimental group not reported Temperature: not reported Humidity: not reported Light/dark cycle: mice exercised in the dark cycle Wheel diameter: 11.5 cm	Breast: syngeneic 4T1 murine breast cancer cells orthotopically transplanted in the dorsal mammary fat pad	Mode: voluntary wheel running Frequency: continuous access to 11.5 cm diameter wheel Duration: 18 days Speed/distance: not reported	Hypoxia: EF5 Vascularisation: MVD by CD31, vascular maturity Blood flow: Perfusion by magnetic resonance imaging (MRI)	Hypoxia: ↓ Vascularisation: ↑ MVD ↑ vessel maturity Blood flow: ↑	
Buss et al. (2018)	*N* = 30–43 female ApoE+/− mice	Housing groups: control group were housed in pairs or threes; experimental group were housed in pairs Temperature: ~22°C Humidity: not reported Light/dark cycle: housed in 12:12-h light/dark cycle Wheel diameter: not reported	Breast: syngeneic EO771 murine medullary breast adenocarcinoma implanted orthotopically into the 4th mammary fat pad	Mode: voluntary wheel running Frequency: continuous wheel access Duration: until tumours reached 600 m^3^ (~17 days) Distance: 10 km/day per pair	Hypoxia: Pimonidazole Vascularisation: MVD by CD31 Blood flow: perfusion by Hoechst 33342 staining	Hypoxia: ↔ Vascularisation: ↔ Blood flow: ↔	Mice were euthanised early if impact on welfare occurred due to ulceration of the tumour (n =8) or suspicion of internal tumours (n=5). One mouse was euthanised before the tumour reached measurable size due to malocclusion.
Buss et al. (2018)	*N* = 30–43 female ApoE+/− mice	Housing groups: control group were housed in pairs or threes; experimental group were housed in pairs Temperature: ~22°C Humidity: not reported Light/dark cycle: housed in 12:12-h light/dark cycle Wheel diameter: not reported	Breast: syngeneic EO771 murine medullary breast adenocarcinoma implanted orthotopically into the 4th mammary fat pad	Mode: voluntary wheel running Frequency: wheel access every 2nd day Duration: until tumours reached 600 m^3^ (~17 days) Distance: 8 km/day per pair	Hypoxia: pimonidazole Vascularisation: MVD by CD31 Blood flow: Pperfusion by Hoechst 33342 staining	Hypoxia: ↔ Vascularisation: ↔ Blood flow: ↔	Mice were euthanised early if impact on welfare occurred due to ulceration of the tumour (*n* =8) or suspicion of internal tumours (*n* = 5). One mouse was euthanised before the tumour reached measurable size due to malocclusion.
Buss et al. (2020)	*N* = 48 C57BL/6 female mice	Housing groups: housed in pairs Temperature: not reported Humidity: not reported Light/dark cycle: housed in 12:12-h light/dark cycle Wheel diameter: not reported	Melenoma and breast: B16-F10 melanoma cells or EO771 breast cells injected subcutaneously into the flank or mammary fat pad	Mode: voluntary wheel running Frequency: continuous access to wheel Duration: until melanoma tumours reached 1000 m^3^ (median 17 days) or breast tumours reached 600 m^3^ (median 21 days) Distance: 8 km/day	Hypoxia: pimonidazole Vascularisation: MVD by CD31 Blood flow: perfusion by Hoechst 33342 staining	Hypoxia: ↔ Vascularisation: ↔ Blood flow: ↔	
Dufresne et al. (2020)	*N* = 17 athymic male nude mice	Housing groups: not reported Temperature: not reported Humidity: not reported Light/dark cycle: housed in 12:12-h light/dark cycle Stimulation for exercise: not reported	Prostate: human prostate cancer PPC-1 cells injected subcutaneously into the dorsal	Mode: treadmill running Frequency: 5×/week Duration: 25–60 min Speed: 18 m/min Slope: 10%	Vascularisation: MVD by CD31	Vascularisation: ↔	
Faustino-Rocha et al. (2016)	*N* = 21 female Sprague-Dawley rats	Housing groups: not reported Temperature: 23 ± 2°C Humidity: 50 ± 10% Light/dark cycle: housed in 12:12-h light/dark cycled; exercised in 12-h dark cycle Stimulation for exercise: not reported	Breast: mammary tumours were induced by a single intraperitoneal administration of the carcinogen agent MNU at a dose of 50 mg/kg	Mode: treadmill running Frequency: 60 min/day for 5×/week Duration: 35 weeks Speed: not reported Slope: not reported	Vascularisation: MVD assessed visually	Vascularisation: ↑	One animal from the MNU exercised group did not adapt to the exercise training and was excluded from the study. During the experiment nine animals died: four animals from the MNU sedentary group (MI = 27%), four animals from the MNU exercised group (MI = 29%) and one animal from the control sedentary (MI = 10%)
Faustino-Rocha et al. (2017)	*N* = 21 female Sprague-Dawley rats	Housing groups: not reported Temperature: 23 ± 2°C Humidity: 50 ± 10% Light/dark cycle: housed in 12:12-h light/dark cycled; exercised in 12-h dark cycle Stimulation for exercise: not reported	Breast: mammary tumours were induced by a single intraperitoneal administration of the carcinogen agent MNU at a dose of 50 mg/kg	Mode: treadmill running Frequency: 60 min/day for 5×/week Duration: 35 weeks Speed: not reported Slope: not reported	Blood flow: Doppler power ultrasound	Blood flow: ↔	One animal from the MNU exercised group did not adapt to the exercise training and was excluded from the study. During the experiment nine animals died: four animals from the MNU sedentary group (MI = 27%), four animals from the MNU exercised group (MI = 29%) and one animal from the control sedentary (MI = 10%) Due to their small size (mammary tumours <1.0 cm were not analysed), only 15 of 28 mammary tumours (54%) from the MNU sedentary group and 11 of 23 (48%) from the MNU exercised group were evaluated by contrast-enhanced US.
Florez-Bedoya et al. (2019)	*N* = 10–14 male nude mice	Housing groups: not reported Temperature: not reported Humidity: not reported Light/dark cycle: not reported Stimulation for exercise: not reported	Pancreatic: patient-derived xenograft of pancreatic ductal adenocarcinoma tumour tissue implanted subcutaneously into the flank	Mode: treadmill running Frequency: 45 min/day for 5 days/week Duration: 4 weeks Speed: 12 m/min Slope: not reported	Vascularisation: MVD by CD31, functional vessels by lectin perfusion	Vascularisation: ↑ MVD ↔ functional vessels	
Isanejad et al. (2016)	*N* = 16 female BALB/c mice	Housing groups: not reported Temperature: not reported Humidity: not reported Light/dark cycle: housed in 12:12-h light/dark cycle; exercised at the end of dark cycle Stimulation for exercise: gentle tap on the tail or hindquarters	Breast: mouse mammary tumour cells MC4-L2 injected into the flank	Mode: treadmill running Frequency: 10–14 min/day for 5 days/week Duration: 5 weeks Speed: 16–18 m/min that increased each week Slope: 0% Stimulus: gentle tap by investigator on the tail or hindquarters	Hypoxia: HIF1α Vascularisation: MVD by CD31	Hypoxia: ↓ Vascularisation: ↓	
Jones et al. (2010)	*N* = 50 female athymic mice	Housing groups: housed individually Temperature: not reported Humidity: not reported Light/dark cycle: exercised in dark cycle Wheel diameter: 11.5 cm	Breast: human mammary adenocarcinoma cell line MDA-MB-231 injected orthotopically into the right dorsal mammary fat pad	Mode: voluntary wheel running Frequency: continuous access to a 11.5 cm diameter wheel Duration: until tumours reached 1500 m^3^ (44 ± 3 days) Distance: ~4 to ~6 km/day	Hypoxia: HIF1α and CAIX Vascularisation: MVD by CD31 Blood flow: perfusion by Hoechst 33342 staining	Hypoxia: HIF1α ↔ CAIX ↔ Vascularisation: ↔ Blood flow: ↑	Histological analysis was only performed on tumours obtained from the 10 animals recording the highest mean exercise running distance and 10 random control animals.
Jones et al. (2012)	*N* = 38 male C57BL/6 mice	Housing groups: housed individually Temperature: 21°C Humidity: 35–45% Light/dark cycle: housed in 12:12-h light/dark cycle Wheel diameter: 11.5 cm	Prostate: transgenic adenocarcinoma of mouse prostate (TRAMP) C-1 cells injected orthotopically into the prostate	Mode: voluntary wheel running Frequency: continuous access to a 11.5 cm diameter wheel Duration: four mice per group were serially killed on days 14, 31 and 36; the remaining 38 mice (exercise, *n* = 18; control, *n* = 20) were killed on day 53. Distance: ~4 to ~6 km/day	Hypoxia: HIF1α Vascularisation: MVD by CD31 Blood flow: perfusion by magnetic resonance imaging (MRI)	Hypoxia: ↑ Vascularisation: ↑ Blood flow: ↑	
McCullough et al. (2013)	*N* = 27 male Copenhagen and nude rats	Housing groups: not reported Temperature: 23°C Humidity: not reported Light/dark cycle: housed in 12:12 light/dark cycle Stimulation for exercise: not reported	Prostate: Dunning R-3327 rat prostate adenocarcinoma cell line in both animal species	Mode: treadmill running Frequency: 60 min/day for 5 days/week Duration: 7 weeks (Copenhagen rats) 5 weeks (nude rats) Speed: 15 m/min Slope: 15°	Hypoxia: EF5 and PO_2_ Vascularisation: patent blood vessels	Hypoxia: EF5 ↓ PO_2_ ↓ Vascularisation: ↔	The duration of intervention in nude rats were shortened by 2 weeks to avoid the potential of tumour size constraints.
Morrell et al. (2019)	*N* = 10–20 male nude mice	Housing groups: not reported Temperature: not reported Humidity: not reported Light/dark cycle: not reported Stimulation for exercise: not reported	Ewing Sarcoma: A673 and TC71 human Ewing Sarcoma cells injected into the backs of mice	Mode: treadmill running Frequency: 45 min/day for 5 consecutive days/week Duration: 2 weeks Speed: 12 m/min Slope: not reported	Hypoxia: HIF1α and CAIX Vascularisation: MVD by CD31, vessel morphology	Hypoxia: A673 tumours - HIF1α ↔ - CAIX↓ TC71 tumours - HIF1α ↔ - CAIX ↔ Vascularisation: ↔ MVD ↓ vessel permeability	
Rafiei et al. (2021)	*N* = 16 female BALB/c mice	Housing groups: not reported Temperature: 22 ± 3°C Humidity: 40–60% Light/dark cycle: housed in 12:12-h cycle Wheel diameter: not reported	Breast: MC4-L2 cancer cells injected subcutaneously	Mode: treadmill running Frequency: 30 min in the first 2 weeks and increasing by 5 min every fortnight Duration: 8 weeks Speed: 14 m/min increasing to 20 m/min in the last 2 weeks Slope: not reported	Hypoxia: HIF1α	Hypoxia: ↓	
Saran et al. (2018)	*N* = 18 American Cancer Institute rats (sex not stated)	Housing groups: not reported Temperature: not reported Humidity: not reported Light/dark cycle: not reported Stimulation for exercise: not reported	Liver: MH-3924A cells were implanted into the liver	Mode: treadmill running Frequency: 770 m for 5 days/week Duration: 6 weeks pretumour implantation, 4 weeks posttumour implantation Speed: not reported	Vascularisation: MVD by CD31	Vascularisation: ↓	
Schadler et al. (2016)	*N* = 10–12 male and female wild-type mice from C57B1/6J	Housing groups: not reported Temperature: not reported Humidity: not reported Light/dark cycle: not reported Stimulation for exercise: not reported	Melanoma and pancreatic: B16F10 melanoma and PDAC-4662 pancreatic ductal adenocarcinoma tumours were injected subcutaneously into the flanks of mice	Mode: treadmill running Frequency: 45 min/day for 5 consecutive days/week Duration: 3 weeks Speed: 12 m/min in mice with PDAC4662 10 m/min in mice with B16F10 Slope: not reported	Vascularisation: MVD by CD31, functional vessels by lectin, vessel length	Vascularisation: ↔ MVD ↑ vessel function ↑ vessel length	
Wakefield et al. (2021)	*N* = 14 female BALB/c mice	Housing groups: housed individually Temperature: not reported Humidity: not reported Light/dark cycle: not reported Wheel diameter: not reported	Breast: EMT6 murine mammary cells were implanted into the 4th mammary	Mode: voluntary wheel running Frequency: continuous access to wheel Duration: until tumours reached 200 mm^3^ (15 ± 4 days) plus an additional 7 days Distance: 9–14 km/day	Hypoxia: HIF1α and HIF2α	Hypoxia: ↓	
Zielinski et al. (2004)	*N* = 137 female BALB/cByJ mice	Housing groups: housed individually Temperature: 23°C Humidity: not reported Light/dark cycle: housed in 12:12-h reverse light cycle; exercised during dark cycle Stimulation for exercise: not reported	Lymphoma: EL-4 lymphoid cells subcutaneously injected in the back behind the neck	Mode: treadmill running Frequency: 3 h or until volitional fatigue (mean time to fatigue 135 ± 25 min) for 7 days/week Duration: 5–14 days Speed: 20–40 m/min Slope: not reported	Vascularisation: MVD by CD31	Vascularisation: ↓	
Clinical studies							
Jones et al. (2013)	*N* = 20 females	Not applicable	Breast	Mode: cycling Frequency: 45 min/day for 3 days/week Duration: 12 weeks Intensity: 55–100% VO_2peak_	Hypoxia: HIF1α Vascularisation: MVD by CD31, cell proliferation Blood flow: PET scan	Hypoxia: ↔ Vascularisation: ↔ MVD ↔ vessel structure Blood flow: ↓	Results for hypoxia and vascularisation were only available for 5 participants per group. Blood flow data was limited due to technical difficulties

All results are *p* < 0.05. *HIF1α*, hypoxia-inducible factor 1-alpha; *HIF2a*, hypoxia-inducible factor 2-alpha; *CAIX*, carbonic anhydrase IX; *MVD*, microvessel density; *PO*_*2*_, partial pressure of oxygen; *MNU*, two *N*-methyl-*N*-nitrosourea

### Hypoxia

**Fig. 4:**
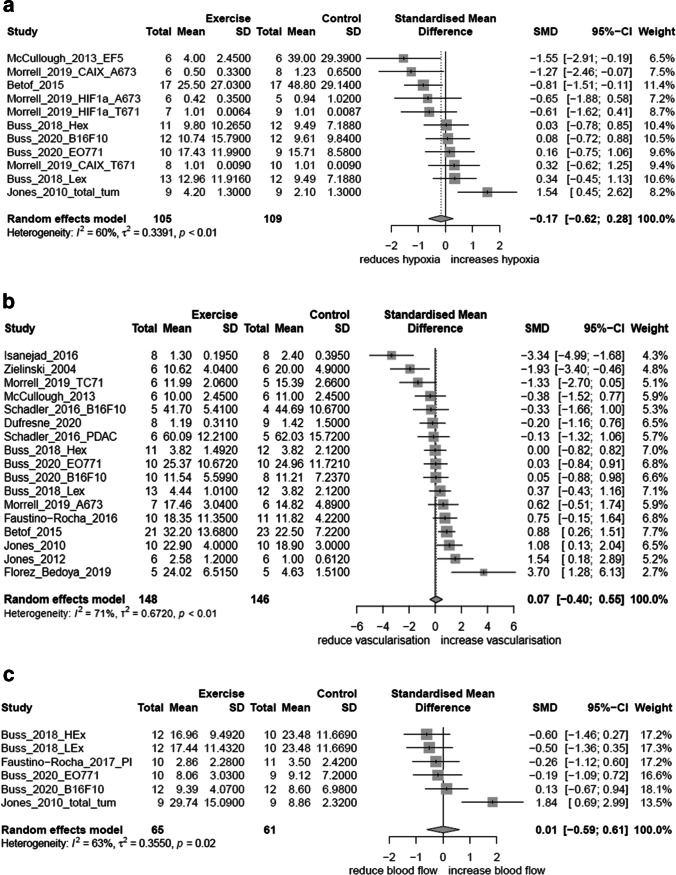
**a** Meta-analysis of preclinical studies investigating the effect of exercise on hypoxia. SD, standard deviation; SMD, standardised mean difference; 95% CI, 95% confidence interval (upper; lower limits). **b** Meta-analysis of preclinical studies investigating the effect of exercise on vascularisation. SD, standard deviation; SMD, standardised mean difference; 95% CI, 95% confidence interval (upper; lower limits). **c** Meta-analysis of preclinical studies investigating the effect of exercise on blood flow. SD, standard deviation; SMD, standardised mean difference; 95% CI, 95% confidence interval (upper; lower limits)

Measures of hypoxia included HIF1α (*n* = 6), EF5 (*n* = 2), pimonidazole (*n* = 2), carbonic anhydrase IX (CAIX) (*n* = 2) and partial pressure of oxygen (PO_2_) (*n* = 1) (Table 2). Some studies assessed multiple measures of hypoxia. Of the 11 studies which assessed hypoxia, six studies reported a decrease in hypoxia [18–23], one study reported an increase [24] and five studies reported no change [23, 25–28]. One study [23] utilised four different datasets, inclusive of two different tumour types and two different measurement methods, finding a decrease and no change in hypoxia within the one paper. Six studies were included in the meta-analysis for hypoxia [18, 20, 23, 25, 26, 28], with three studies having multiple measures ﻿analysed [23, 25, 26]. Four studies were excluded from the meta-analysis due to implausible physiological values or lack of continuous mean data [19, 22, 24]. Overall, there was no significant effect of aerobic exercise on hypoxia (SMD = −0.17; 95% CI = −0.62, 0.28; *I*^2^ = 60%) (Fig. 4a).

### Vascularisation

Measures of vascularisation included microvessel density (*n* = 14), functional vessels (*n* = 2), vessel length (*n* = 1), patent vessels (*n* = 1) and indicators for vessel function and structure (*n* = 3) (Table 2). Four studies reported an increase in microvessel density [18, 24, 29, 30], three studies reported a decrease in microvessel density [19, 31, 32] and eight studies reported no change in microvessel density [20, 23, 25–28, 33, 34]. Twelve studies were included in the meta-analysis for vascularisation [18–20, 23–30, 33, 34], with four studies having multiple measures [23, 25, 26, 34]. One study was excluded due to no baseline data [32]. One study [31] was excluded from the presented forest plot for vascularisation due to being an outlier, resulting in reduced legibility of the remainder of the meta-analysis results (SMD = 0.07; 95% CI = −0.51, 0.64; *I*^2^ = 79%) (full plot available in Supplemental Figure 1). The exclusion made no difference in the overall effect size. Overall, there was no effect of aerobic exercise on vascularisation (SMD = 0.07; 95% CI = −0.40, 0.55; *I*^2^ = 71%) (Figure 4b).

### Blood flow

Of the seven studies that evaluated blood flow, three reported an increase [18, 24, 28], one reported a decrease [27] and three reported no change [25, 26, 35] (Table 2). Four studies were included in the meta-analysis for blood flow [25–28, 35], with two studies having multiple measures, including blood flow and perfusion [25, 26]. One study was excluded from the meta-analysis due to no quantitative data reported, and another study was excluded as there were no mean data [18, 24]. Overall, there was no significant effect of aerobic exercise on blood flow (SMD = 0.01; 95% CI = −0.59, 0.61; *I*^2^ = 63%) (Figure 4c).

### Sub-group analysis

Given the heterogeneity of methodology in the studies included in this review, the total meta-analysis is limited. In an attempt to review the data taking into account some of these methodological differences, we performed sub-group analyses for animals, species, aerobic exercise mode, tumour location and study duration. However, it should be noted that these sub-groups included large heterogeneity, such as in exercise mode (for example, treadmill exercise dose). We therefore performed sub-group analyses using broad categories to analyse the data. The results from the sub-analyses showed no significant effect in any of the analyses. Sub-group analyses can be found in the Supplemental materials (Supplemental Figures 2a–e, 3a–e and 4a–e).

## Discussion

The findings from this systematic review and meta-analysis demonstrate that among 17 preclinical studies and one clinical study, aerobic exercise training had no significant effect on tumour hypoxia, vascularisation or blood flow. To our knowledge, this is the only systematic review exploring the effects of hypoxia, vascularisation and blood flow on all three of these related outcomes. Concerningly, there was a high risk for attrition bias in the preclinical studies, and most other domains of bias were of unclear risk. Of the 18 studies included in this review, 17 were published within the last decade, suggesting that there is a growing interest in this area. Although the preclinical body of evidence is growing, the synthesis of data is limited by the variability in animal type and study methodology. The measurement of the outcomes reported herein is possible in a human population, as evidenced in Jones et al. (2013). Therefore, future knowledge gains may be made by exploring these effects in humans for translation to clinical practice.

We anticipated that aerobic exercise would drive an association between our outcome measures; that aerobic exercise training would result in an increase in vascularisation that would cause increased blood flow and consequently decreased hypoxia, representing beneficial changes in the tumour microenvironment with the potential to reduce tumour growth and improve patient prognosis. This assumption has been based on the literature, exemplified by recent narrative reviews [36, 37]. However, the expected pattern of change was not found in this review, with studies reporting aerobic exercise-induced decreases and increases in all outcome measures and combinations of decreases and increases that do not support our assumption. Although our findings contrast with recent narrative reviews, our conclusions are supported by the systematic inclusion of all available data.

In the period between registration and completion of this review, Esteves et al. [38] published a systematic review and meta-analysis that included one of the outcomes we assessed: vascularisation. The authors found an effect of aerobic exercise on vascularisation, which is in conflict with the findings from this review. We have described a number of concerns with the methodology, data extraction and data analysis of their review elsewhere [39]. The current comprehensive review and meta-analysis uses a more appropriate statistical analysis recommended for preclinical studies [40–42] and includes all available studies, providing a more accurate evaluation of the current literature.

Contrary to our expectations, we found that there were inconsistent findings for all explored outcomes. We expected to see decreases in hypoxia; however, of the 11 studies which assessed hypoxia, one [24] found an unexpected increase and five [23, 25–28] found no change. We expected increased vascularisation; however, three of 15 studies [19, 31, 32] showed a reduction and eight [20, 23, 25–28, 33, 34] showed no change. Our expectation of increased blood delivery was also not met; three studies demonstrated no change [25, 26, 35], and one study demonstrated a decrease [27] in blood flow. These unexpected results in hypoxia, vascularisation and blood flow may be a result of many factors, including experimental design differences as well as intra-tumoural factors. For example, physiological mechanisms that may have contributed to these results include angiogenesis development and/or decreased inflammatory immune cells. Aerobic exercise induces the formation of new blood vessels through regulatory factors such as increases in VEGF, which promotes angiogenesis [24]. Although angiogenesis may increase microvessel density and subsequent blood flow to tumours, the dysfunction in tumour blood vessels (specifically leaky blood vessels and low permeability) may mean that this is not translated to changes in hypoxia if oxygen diffusion is poor [9, 10, 31, 40]. Furthermore, tumour blood vessels are heterogeneous in distribution, leading to inconsistent oxygen supply [8], which may mean areas of increased hypoxia within the tumour despite potential increases in overall blood flow through increased microvessel density. Therefore, a single hypoxia measurement may not represent the level of hypoxia elsewhere in the tumour. Immune cell’s contribution to angiogenesis has also been suggested to be altered by aerobic exercise training. For example, inflammatory macrophages in tumours have been shown to have reduced activation after aerobic training [32]. This type of reduced inflammatory activation may downregulate angiogenesis and reduced microvessel density, resulting in less blood delivery and increased hypoxia [32].

Variability in study methodology and quality can create significant bias in the results. Features of poor study methodology, such as lack of reporting, heterogeneity of baseline characteristics and external influences such as housing (in preclinical research), aerobic exercise outside of the intervention and significant missing data, can largely impact results both within studies and when comparing studies. The risk of bias results from this review should be used to guide future preclinical and clinical methodology to better compare outcome measures across studies. Housing was poorly reported, which is important for social stress effects [43]. Additionally, the impact of stress on animals, such as stimulation for forced aerobic exercise, is particularly significant in cancer outcomes, whereby it has been postulated that the immune response plays a part in the development of tumour vasculature to promote microvessel density and tumour growth in mice [44, 45]. The characteristics of aerobic exercise were poorly reported across studies. Six studies [18, 22, 24–26, 28] used voluntary wheel running, which suffers from a lack of control of intensity and, therefore, dose. Seven papers included a familiarisation period of aerobic exercise for an average of 18 days [18–20, 29, 32, 33, 35], whereby subjects would perform additional aerobic exercise after tumour induction but prior to the aerobic exercise intervention. Exercise duration includes a combination of familiarisation plus intervention, which further complicates the dose of aerobic exercise delivered, particularly in preclinical models. In the current analysis, we saw heterogeneity in animal type and species (14 mice studies with five different species and four rat studies with three different species), making comparison of results between papers problematic and suitability for human models debateable [46].

Tumour type and location likely play a considerable role in determining the effects of aerobic exercise on blood flow. Host tissue is anatomically and functionally different, and thus features of the tumour microenvironment that influence blood flow may also differ based on tumour location. Therefore, it may not be equal to compare tumour type and location. For example, tumours located in host tissue that has a high blood supply at rest, such as the brain, are more likely to use vascular co-option, which encourages greater tumour vascularisation [47]. Furthermore, vasculogenic mimicry occurs in some tumour types, such as melanoma and breast, and can also increase blood supply to and within the tumour [48]. In preclinical models, tumours are induced orthotopically (tumours that are injected into the corresponding host tissue) or ectopically (tumours injected subcutaneously into a different host tissue). Garcia and colleagues [49] directly compared ectopic prostate tumours placed subcutaneously with orthotopic prostate tumours and found increased blood flow in orthotopic tumours after aerobic exercise, suggesting that tumour location plays an important role irrespective of tumour type. We included 13 papers that investigated orthotopically injected tumours and two papers that investigated subcutaneously injected tumours, and we observed no difference in blood flow after aerobic exercise in orthotopic compared to subcutaneous tumours, which is contrary to the results of Garcia et al [49]. However, a majority of the papers in this systematic review investigated breast tumours, whilst Garcia et al. [49] investigated prostate tumours. Therefore, further research needs to be conducted to determine if tumour type, in addition to tumour location, influences blood flow.

Whilst our findings do not favour aerobic exercise training in terms of tumour hypoxia, vascularisation and blood flow, several systematic reviews and meta-analyses have found beneficial effects of aerobic exercise training for tumour growth and development [44, 50]. Given this contrast, we briefly examined tumour growth outcomes, which were reported in 13 studies included in this review [18, 21, 22, 24–26, 28–34]. Of these, there were inconsistent results in tumour growth; one reported increased growth, four showed decreased growth and nine showed no change in tumour size. One study [34] showed both a decrease in one tumour type and an increase in another tumour type. We observed similar inconsistent patterns of change in hypoxia, vascularisation and blood flow even with studies showing the same tumour growth changes. However, it should be noted that tumour growth was not a primary outcome of this review and the reported findings are descriptive only. As the studies included in this review did not mirror the results from the two published systematic reviews on tumour growth, we believe that the mechanisms of action for aerobic exercise on tumour growth are complex and not well described by this literature. It is possible that the mechanisms of aerobic exercise effects could involve interactions between hypoxia, vascularisation and blood flow; however, more consistent data, methodology and reporting are necessary to further explore these associations. It is also likely that other systemic or intra-tumoural factors, such as inflammation, immune responses, myokine signalling and endocrine responses and adaptations, impact tumour growth [44, 51, 52].

### Limitations

Despite using author-reported results, we found that there were two instances where there were differences in the significance of findings between author-reported results and the meta-analysis calculation. Our careful review and meta-analysis showed inconsistencies within a paper, which were confirmed with the authors but may have been the cause of the different outcomes in the analysis. Subsequently, it is possible that the conclusions of this review would differ without the inclusion of a meta-analysis calculation. Another limitation is that although we conducted sub-group analyses, the results were limited due to poor reporting and heterogeneous study design. Furthermore, our meta-analysis examined one mode of exercise, aerobic exercise only, which does not fully represent the effect of all exercise modes, including resistance exercise.

## Conclusion

Among the studies included in this systematic review, aerobic exercise training did not have an effect on tumour hypoxia, vascularisation or blood flow. However, there was great methodological heterogeneity, which may have contributed to the inconsistent findings. Future preclinical studies need improved study design and reporting to provide deeper insights into the complex interactions between hypoxia, vascularisation and blood flow. Furthermore, improved reporting and subsequent analysis of aerobic exercise parameters in future studies, specifically intensity and dosage, will give rise to a better understanding of the effects of aerobic exercise on tumour microenviroment for translation to clinical studies. Given the established benefits of aerobic exercise in reducing cancer burden, understanding the mechanisms is an important step towards designing the most efficacious interventions and best practise for translation to clinical studies.

## Supplementary Information

ESM 1 (DOCX 1329 kb)

## Data Availability

The datasets from this review are available from the corresponding author on reasonable request.
