# Supplementary material for: The effect of aerobic exercise on tumour blood delivery: a systematic review and meta-analysis
Source: Support Care Cancer. 2022 Jun 2;30(11):8637–53. doi: 10.1007/s00520-022-07132-0 (PMC9633495; doi:10.1007/s00520-022-07132-0)
Supplement: Supplementary file 1 — (DOCX 1329 kb) [file 520_2022_7132_MOESM1_ESM.docx]

**The effect of aerobic exercise on tumour blood delivery: a systematic review and meta‐analysis**

Supplementary material

| Author, year | Participants | Cancer type | Intervention | Outcome measure: method of measure | Hypoxia | Vascularisation (MVD results only) | Blood flow |
| --- | --- | --- | --- | --- | --- | --- | --- |
| Preclinical studies | | | | | | | |
| Betof et al., 2015 | Female immunocompetent BALB/c mice | Breast | Voluntary wheel running  for 18 days | Hypoxia: EF5  Vascularisation: MVD by CD31, vascular maturity  Blood flow: Perfusion by magnetic resonance imaging (MRI) | - Control: n=17; mean = 48.80 (95% CI = 35.5-63.2) - Exercise: n=17; mean = 25.5 (95% CI = 12.7-38.4) | Control: n=23; mean = 22.5 (95% CI = 19.6-25.5)  Exercise: n=21; mean = 32.2 (95% CI = 26.3-38.0) | - No quantitative data shown |
| Buss et al., 2018 | Female ApoE+/- mice | Breast | Voluntary wheel running until tumours reached 600m^3^ (~17 days) | Hypoxia: Pimonidazole  Vascularisation: MVD by CD31  Blood flow: Perfusion by Hoechst 33342 staining | Control: n=12; mean = 9.49; SEM = 2.075  Exercise: n=13; mean = 12.96 (SEM = 3.305) | Control: n=12; mean = 3.82; SEM = 0.635  Exercise: n=11; mean = 3.82; SEM = 0.45 | - Control: n=10; mean = 23.48; SEM = 3.69   Exercise: n=12; mean = 16.965; SEM = 2.74 |
| Buss et al., 2018 | Female ApoE+/- mice | Breast | Voluntary wheel running  every 2^nd^ day  until tumours reached 600m^3^ (~17 days) | Hypoxia: Pimonidazole  Vascularisation: MVD by CD31  Blood flow: Perfusion by Hoechst 33342 staining | Control: n=12; mean = 9.49; SEM = 2.075  Exercise: n=12; mean = 9.8; SEM = 3.095 | Control: n=12; mean = 3.82; SEM = 0.635  Exercise: n=13; mean = 4.44; SEM = 0.305 | - Control: n=10; mean = 23.48; SEM = 3.69   Exercise: n=12; mean = 17.44; SEM = 3.3 |
| Buss et al., 2020 | C57BL/6 female mice | Melenoma and breast | Voluntary wheel running  until melanoma tumours reached 1000m^3^ (median 17 days) or breast tumours reached 600m^3^ (median 21 days) | Hypoxia: Pimonidazole  Vascularisation: MVD by CD31  Blood flow: Perfusion by Hoechst 33342 staining | B16F10 tumours  Control: n=12; mean = 9.61 (95% CI = 3.86-15.0)  Exercise: n=12; mean = 10.745 (95% CI = 1.42-19.29)  EO771 tumours  Control: n=9; mean = 15.71 (95% CI = 10.15-21.36)  Exercise: n=10; mean = 17.43 (95% CI = 10.15-25.01) | B16F10 tumours  Control: n=8; mean = 11.21 (95% CI = 6.195-16.226)  Exercise: n=10; mean = 11.545 (95% CI = 8.08-15.02)  EO771 tumours  Control: n=10; mean = 24.965 (95% CI = 17.7-32.23)  Exercise: n=10; mean = 25.37 (95% CI = 18.755-31.985) | - B16F10 tumours - Control: n=12; mean = 8.595 (95% CI = 4.78-12.68)   Exercise: n=12; mean = 9.39 (95% CI = 6.22-10.83)  EO771 tumours  Control: n=9; mean = 9.12 (95% CI = 4.41-13.82)  Exercise: n=10; mena = 8.06 (95% CI = 6.1-9.85) |
| Dufresne et al., 2020 | Athymic male Nude mice | Prostate | Treadmill running 5 times/week for 25-60 minutes at 18m/min with 10% slope for 2 weeks | Vascularisation: MVD by CD31 |  | Control: n=9; mean = 1.42; SEM = 0.5  Exercise: n=8; mean = 1.185; SEM = 0.11 |  |
| Faustino-Rocha et al., 2016 | Female Sprague-Dawley rats | Breast | Treadmill running 5x/week for 60 minutes for 35 weeks | Vascularisation: MVD assessed visually |  | Control: n=11; mean = 11.82; SEM = 1.09  Exercise: n=10; mean = 18.35; SEM = 2.93 |  |
| Faustino-Rocha et al., 2017 | Female Sprague-Dawley rats | Breast | Treadmill running 5x/week for 60 minutes for 35 weeks | Blood flow: Doppler power ultrasound |  |  | - PI - Control: n=11; mean = 0.87; SEM = 0.1 - Exercise: n=10; mean = 0.79; SEM = 0.08 |
| Florez-Bedoya et al., 2019 | Male Nude mice | Pancreatic | Treadmill running for 5 days/week for 45 minutes at 12m/min for 4 weeks | Vascularisation: MVD by CD31, functional vessels by lectin perfusion |  | Control: n=5; mean = 4.63; SD = 1.51  Exercise: n=5; mean = 24.025; SD = 6.515 |  |
| Isanejad et al., 2016 | Female BALB/c mice | Breast | Treadmill running 10-14 mins/day for 5 days/week at 6-18m/min that increased each week for  5 weeks | Hypoxia: HIF1α  Vascularisation: MVD by CD31 | - Control: n=8; mean fold change = 1; SD = 0 - Exercise: n=9; mean fold change = 0.215; SD = 0.01 | Control: n=8; mean fold change = 2.4; SD = 0.395   - Exercise: n=8; mean fold change = 1.3; SD = 0.195 |  |
| Jones et al., 2010 | Female Aythmic mice | Breast | Voluntary wheel running until tumours reached 1500m^3^ (44±3 days) | Hypoxia: HIF1α and CAIX  Vascularisation: MVD by CD31  Blood flow: Perfusion by Hoechst 33342 staining | Total tumour  HIF1α  Control: n=9-10; mean = 2.1; SD = 1.3  Exercise: n=9-10; mean = 4.2; SD = 1.3  CAIX  No data shown | Control: n=10; mean = 18.9; SD = 3.0  Exercise: n=10; mean = 22.9; SD = 4.0 | - Control: n=9-10; mean = 3; SD = 0.45   Exercise: n=9-10; mean = 7.4; SD = 1.8 |
| Jones et al., 2012 | Male C57BL/6 mice | Prostate | Voluntary wheel running for 53 days | Hypoxia: HIF1α  Vascularisation: MVD by CD31  Blood flow: Perfusion by magnetic resonance imaging (MRI) | Control: n=6-10; mean = -7.69; SEM = 4.62  Exercise: n=6-10; mean = 14.205; SEM = 4.44 | Control: n=6-10; mean = 1; SEM = 0.25  Exercise: n=6-10; mean = 2.585; SEM = 0.49 | - Control: n=5-6; median = 1.83   Exercise: n=5-6; median = 2.125 |
| McCullough et al., 2013 | Male Copenhagen and Nude rats | Prostate | Treadmill running for 5 days/week for 60 minutes at 15m/min with 15 degrees slope for 7 weeks (Copenhagen rats) and 5 weeks (Nude rats) | Hypoxia: EF5 and PO_2_  Vascularisation: Patent blood vessels | EF5  Control: n=6; mean = 39; SEM = 12  Exercise: n=6; mean = 4; SEM = 1  PO_2_  Control: n=6; mean = 6; SEM = 0.3  Exercise: n=9; mean = 12.2; SEM = 1 | Control: n=6; mean = 11; SEM = 1  Exercise: n=6; mean = 10; SEM = 1 |  |
| Morrell et al., 2019 | Male Nude mice | Ewing Sarcoma | Treadmill running for 5 days/week for 45 minutes at 12m/min for 2 weeks | Hypoxia: HIF1α and CAIX  Vascularisation: MVD by CD31, vessel morphology | - A673 tumours - HIF1α   Control: n=5; mean = 0.9396; SEM = 0.4542  Exercise: n=6; mean = 0.4218; SEM = 0.1432  CAIX  Control: n=8; mean = 1.231; SEM = 0.2314  Exercise: n=6; mean = 0.5; SEM = 0.1353   - TC71 tumours - HIF1α ↔ - Control: n=9; mean = 1.011; SEM = 0.0029 - Exercise: n=7; mean = 1.006; SEM = 0.0024   CAIX  Control: n=10; mean = 1.01; SEM = 0.0028  Exercise: n=8; mean = 1.013; SEM = 0.0032 | A673 tumours  Control: n=6; mean = 14.82; SEM = 1.995   - Exercise: n=7; mean = 17.46; SEM = 1.15   TC71 tumours  Control: n=5; mean = 15.39; SEM = 1.19  Exercise: n=6; mean = 11.99; SEM = 0.84 |  |
| Rafiei et al., 2021 | Female BALB/c mice | Breast | Treadmill running for 5 days/week for 30-45 minutes at 14-20m/min for 8 weeks | Hypoxia: HIF1α | - Control: n=8; mean fold change = 0.95; SD = 0.17 - Exercise: n=8; mean fold change= 1.93; SD = 0.54 |  |  |
| Saran et al., 2018 | American Cancer Institute rats | Liver | Treadmill running for 5 days/week for 770m for 6 weeks | Vascularisation: MVD by CD31 |  | Control: n=9; mean = 80.565; SD = 2.05   - Exercise: n=9; mean = 30.3; SD = 2.245 |  |
| Schadler et al., 2016 | Male and female wild-type mice from C57B1/6J | Melanoma and pancreatic | Treadmill running for 5 days/week for 45 minutes at 12m/min (PDAC4662) or 10m/min (B16F10) for 3 weeks | Vascularisation: MVD by CD31, functional vessels by lectin, vessel length |  | B16F10 tumours  Control: n=4; mean = 44.685; SEM = 5.335  Exercise: n=5; mean = 41.705; SME = 2.42  PDAC tumours  Control: n=5; mean = 62.03; SEM = 7.03  Exercise: n=6; mean = 60.095; SEM = 4.985 |  |
| Wakefield et al., 2021 | Female BALB/c mice | Breast | Voluntary wheel running until tumours reached 200mm^3^ (15±4 days) plus an additional 7 days | Hypoxia: HIF1α and HIF2α | HIF1a  Control: n=7; median = 0.94  Exercise: n=7; median = 0.64  HIF2a  Control: n=7; median = 0.98  Exercise: n=7; median = 0.83 |  |  |
| Zielinski et al., 2004 | Female BALB/cByJ mice | Lymphoma | Treadmill running for 7 days/week for 3 hours or until volitional fatigue at 20-40m/min for 5-14 days | Vascularisation: MVD by CD31 |  | - Unable to calculate due to no baseline data shown |  |
| Clinical study | | | | | | | |
| Jones et al., 2013 | Females | Breast | Cycling for 3 days/week for 45 mins/day at 55-100% VO_2peak_ for 12 weeks | Hypoxia: HIF1α  Vascularisation: MVD by CD31, cell proliferation  Blood flow: PET scan | Data not shown | Data not shown | Data not shown; tumour blood flow reduced by 38% |

Supplementary table 1: raw data from studies

SD = standard deviation; SEM = standard error of the mean; 95% CI = 95% confidence interval; HIF1α = hypoxia-inducible factor 1-alpha; HIF2a = hypoxia-inducible factor 2-alpha; CAIX = carbonic anhydrase IX; MVD = microvessel density; PO_2_ = partial pressure of oxygen; MNU = two N-methyl-N-nitrosourea


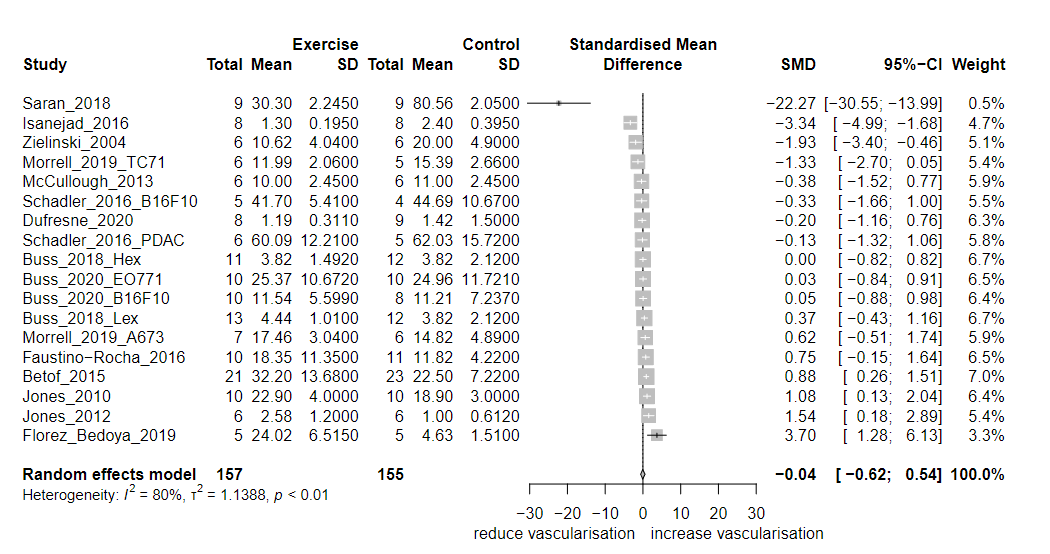
 Supplementary figure 1: meta-analysis of vascularisation including Saran et al paper.

SD = standard deviation; SMD = standardised mean difference; 95% CI = 95% confidence interval (upper; lower limits)


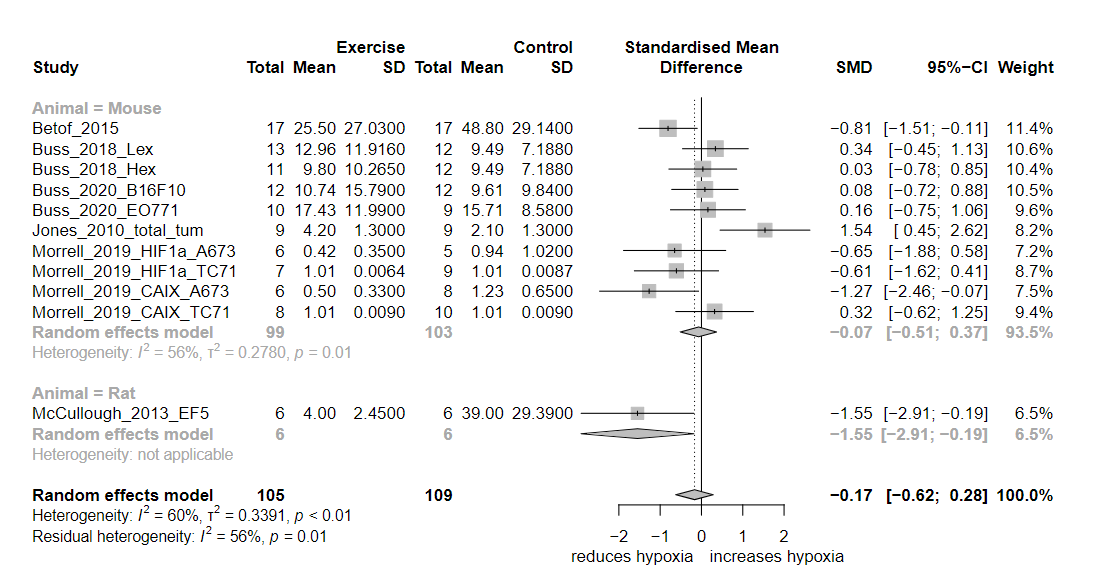


Supplementary figure 2a: sub-group analysis by animal for hypoxia

SD = standard deviation; SMD = standardised mean difference; 95% CI = 95% confidence interval (upper; lower limits)


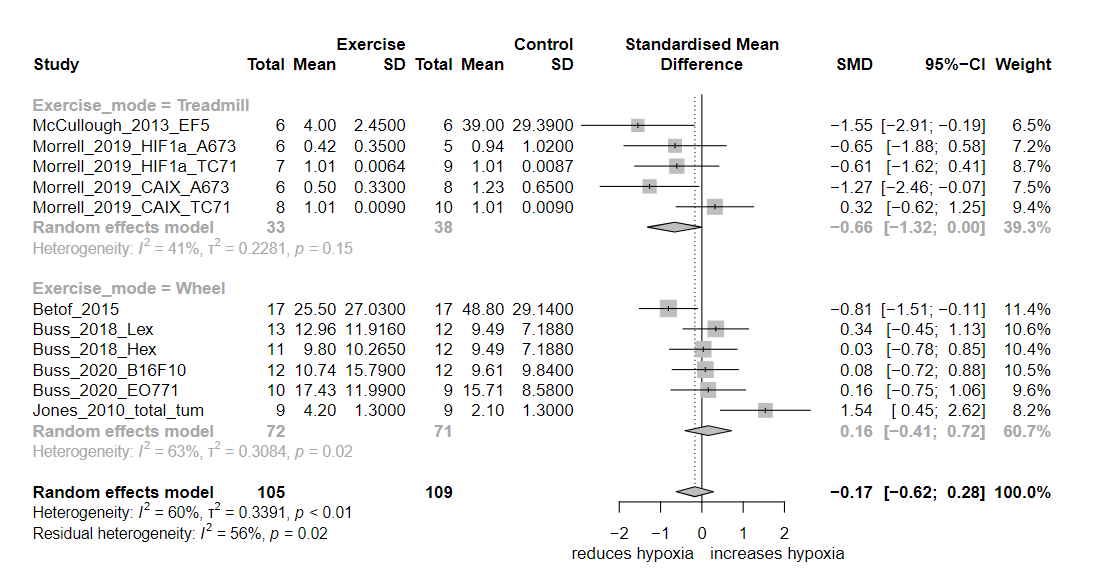
Supplementary figure 2b: sub-group analysis by exercise mode for hypoxia

SD = standard deviation; SMD = standardised mean difference; 95% CI = 95% confidence interval (upper; lower limits)


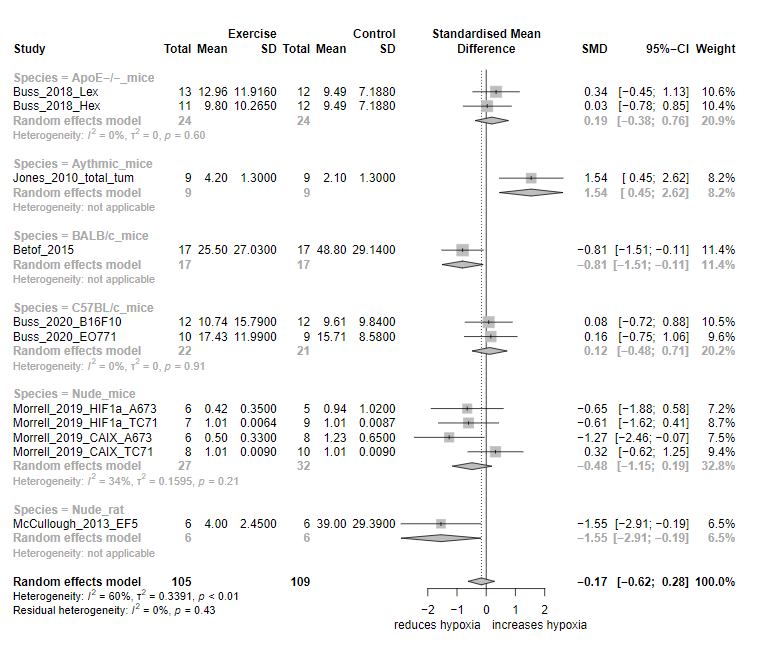


Supplementary figure 2c: sub-group analysis by animal species for hypoxia

SD = standard deviation; SMD = standardised mean difference; 95% CI = 95% confidence interval (upper; lower limits)


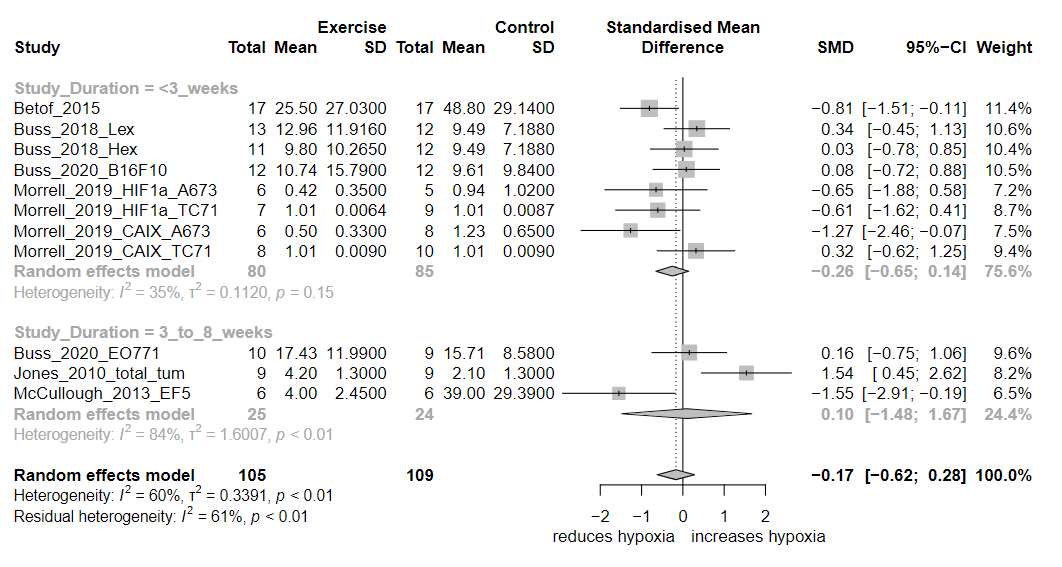
Supplementary figure 2d: sub-group analysis by study duration for hypoxia

SD = standard deviation; SMD = standardised mean difference; 95% CI = 95% confidence interval (upper; lower limits)

Supplementary figure 2e: sub-group analysis by tumour type for hypoxia
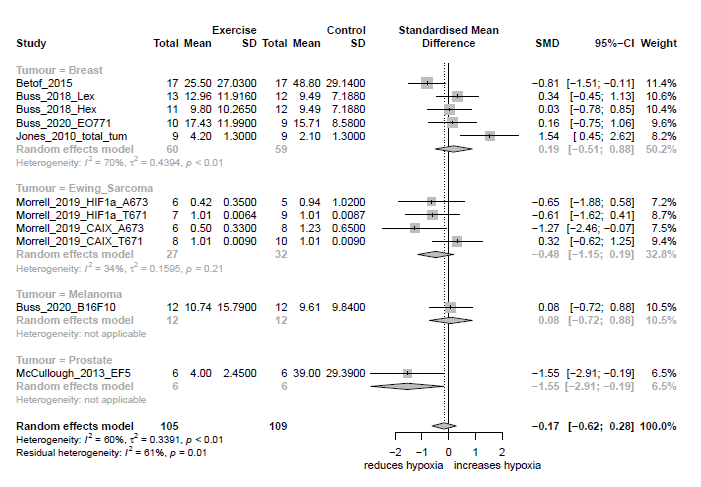


SD = standard deviation; SMD = standardised mean difference; 95% CI = 95% confidence interval (upper; lower limits)


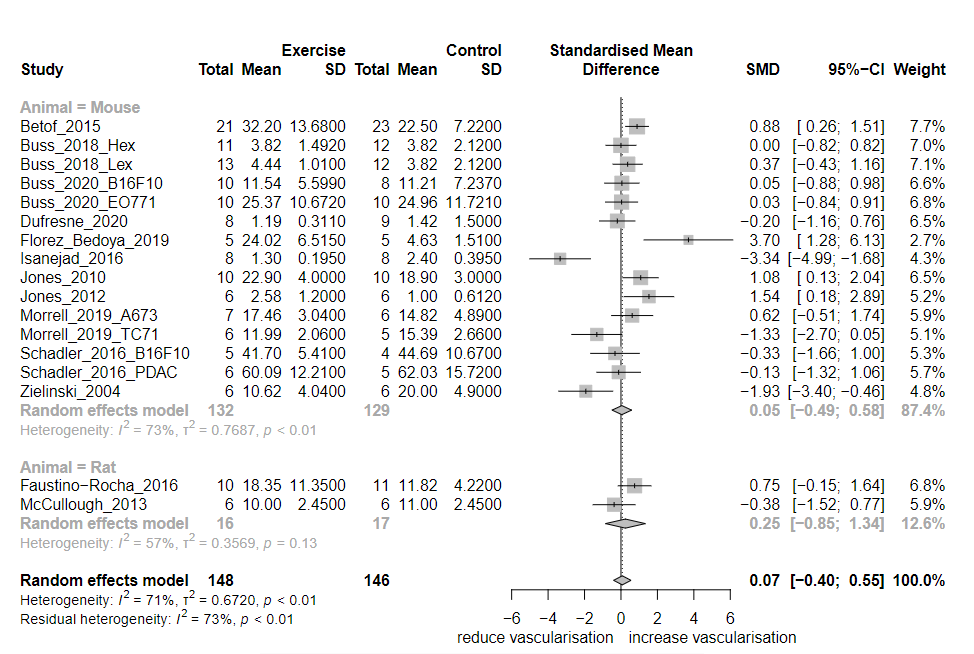
Supplementary figure 3a: sub-group analysis by animal for vascularisation

SD = standard deviation; SMD = standardised mean difference; 95% CI = 95% confidence interval (upper; lower limits)


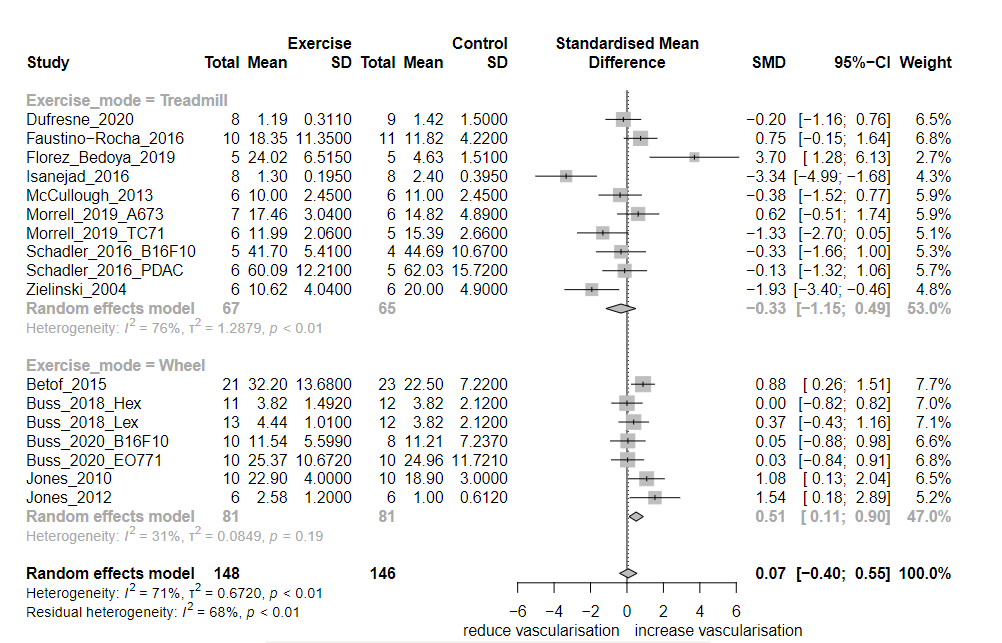
Supplementary figure 3b: sub-group analysis by exercise mode for vascularisation

SD = standard deviation; SMD = standardised mean difference; 95% CI = 95% confidence interval (upper; lower limits)


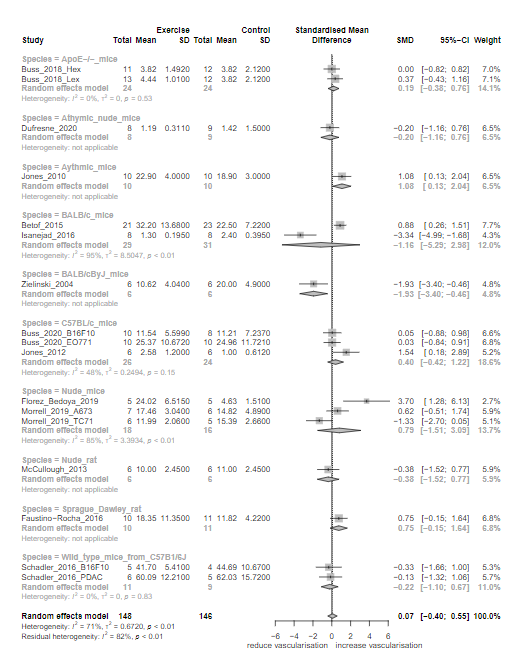


Supplementary figure 3c: sub-group analysis by animal species for vascularisation

SD = standard deviation; SMD = standardised mean difference; 95% CI = 95% confidence interval (upper; lower limits)


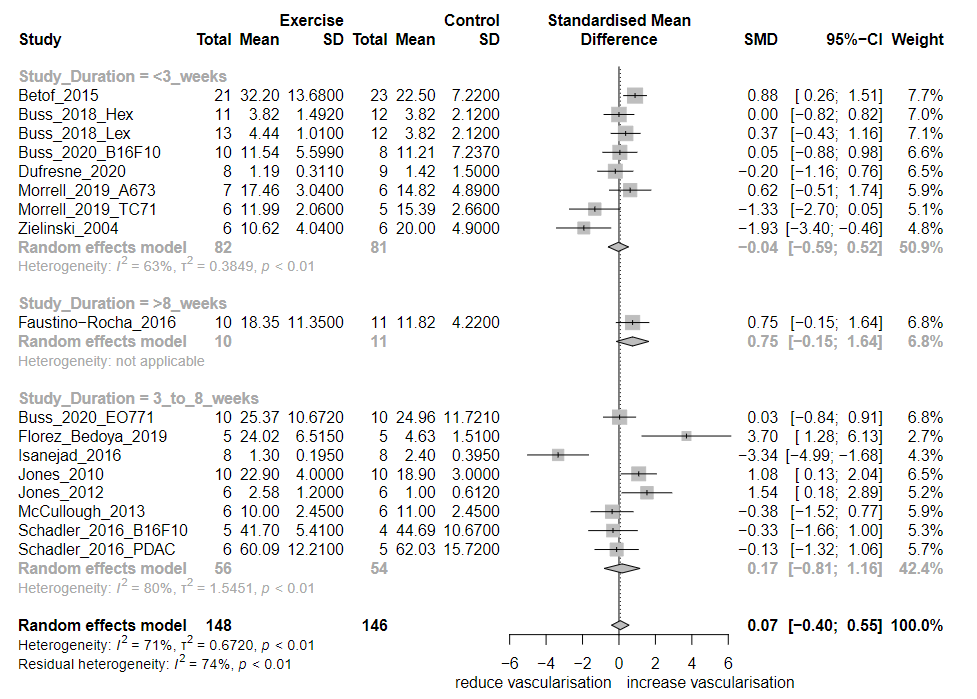
Supplementary figure 3d: sub-group analysis by study duration for vascularisation

SD = standard deviation; SMD = standardised mean difference; 95% CI = 95% confidence interval (upper; lower limits)


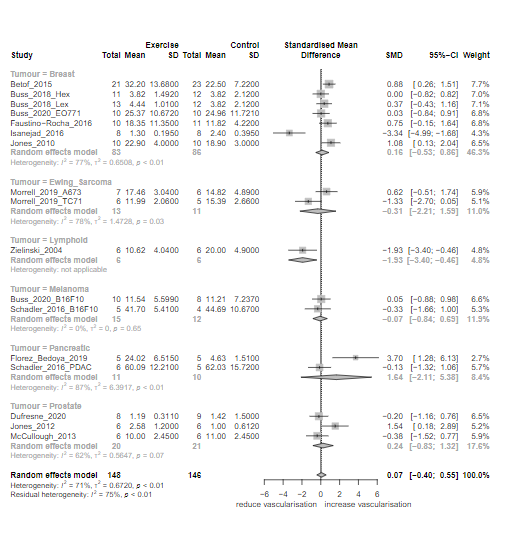


Supplementary figure 3e: sub-group analysis by tumour type for vascularisation

SD = standard deviation; SMD = standardised mean difference; 95% CI = 95% confidence interval (upper; lower limits)


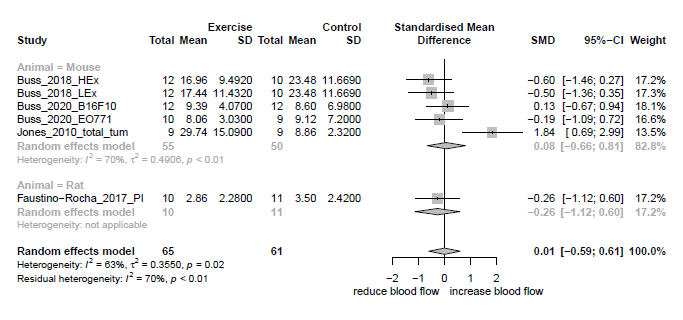


Supplementary figure 4a: sub-group analysis by animal for blood flow

SD = standard deviation; SMD = standardised mean difference; 95% CI = 95% confidence interval (upper; lower limits)


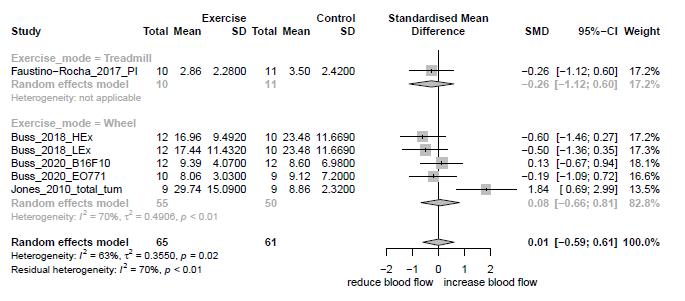


Supplementary figure 4b: sub-group analysis by exercise mode for blood flow

SD = standard deviation; SMD = standardised mean difference; 95% CI = 95% confidence interval (upper; lower limits)


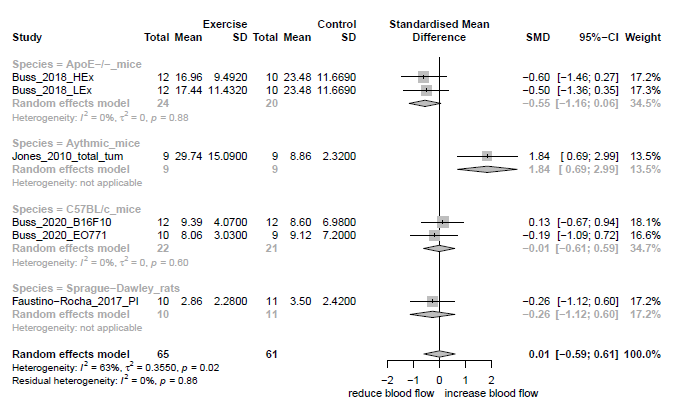
Supplementary figure 4c: sub-group analysis by animal species for blood flow

SD = standard deviation; SMD = standardised mean difference; 95% CI = 95% confidence interval (upper; lower limits)

Supplementary figure 4d: sub-group analysis by study duration for blood
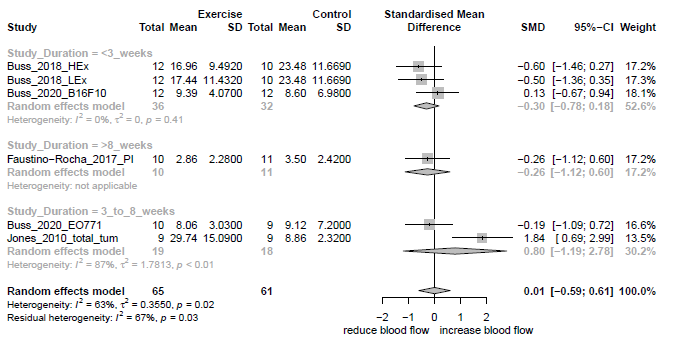
flow

SD = standard deviation; SMD = standardised mean difference; 95% CI = 95% confidence interval (upper; lower limits)


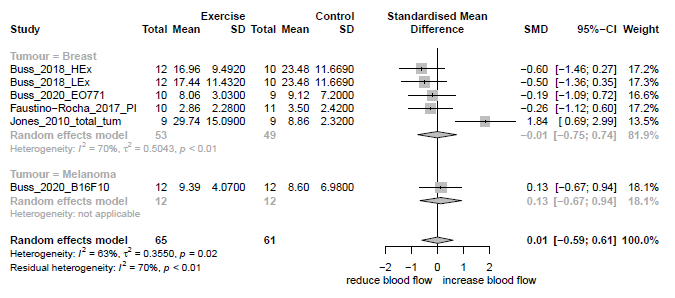


Supplementary figure 4e: sub-group analysis by tumour type for blood flow

SD = standard deviation; SMD = standardised mean difference; 95% CI = 95% confidence interval (upper; lower limits)
